# Atto488-Agitoxin 2—A Fluorescent Ligand with Increased Selectivity for Kv1.3 Channel Binding Site

**DOI:** 10.3390/bioengineering9070295

**Published:** 2022-07-01

**Authors:** Kristina R. Denisova, Nikita A. Orlov, Sergey A. Yakimov, Mikhail P. Kirpichnikov, Alexey V. Feofanov, Oksana V. Nekrasova

**Affiliations:** 1 Faculty of Biology, Lomonosov Moscow State University, 119234 Moscow, Russia; tina.denisova2000@gmail.com (K.R.D.); n.orlov858@yandex.ru (N.A.O.); kirpichnikov@inbox.ru (M.P.K.); 2 Shemyakin-Ovchinnikov Institute of Bioorganic Chemistry, Russian Academy of Sciences, 117997 Moscow, Russia; sa-yakimov@yandex.ru (S.A.Y.); onekrasova@ibch.ru (O.V.N.)

**Keywords:** agitoxin 2, Kv1.3 channel, pore blocker, fluorescent ligand, affinity, KcsA, hybrid channels

## Abstract

Fluorescently labeled peptide blockers of ion channels are useful probes in studying the localization and functioning of the channels and in the performance of a search for new channel ligands with bioengineering screening systems. Here, we report on the properties of Atto488-agitoxin 2 (A-AgTx2), a derivative of the Kv1 channel blocker agitoxin 2 (AgTx2), which was N-terminally labeled with Atto 488 fluorophore. The interactions of A-AgTx2 with the outer binding sites of the potassium voltage-gated Kv1.x (x = 1, 3, 6) channels were studied using bioengineered hybrid KcsA–Kv1.x (x = 1, 3, 6) channels. In contrast to AgTx2, A-AgTx2 was shown to lose affinity for the Kv1.1 and Kv1.6 binding sites but to preserve it for the Kv1.3 site. Thus, Atto488 introduces two new functionalities to AgTx2: fluorescence and the selective targeting of the Kv1.3 channel, which is known for its pharmacological significance. In the case of A-AgTx2, fluorescent labeling served as an alternative to site-directed mutagenesis in modulating the pharmacological profile of the channel blocker. Although the affinity of A-AgTx2 for the Kv1.3 binding site was decreased as compared to the unlabeled AgTx2, its dissociation constant value was within a low nanomolar range (4.0 nM). The properties of A-AgTx2 allow one to use it for the search and study of Kv1.3 channel blockers as well as to consider it for the imaging of the Kv1.3 channel in cells and tissues.

## 1. Introduction

**Figure 1:**
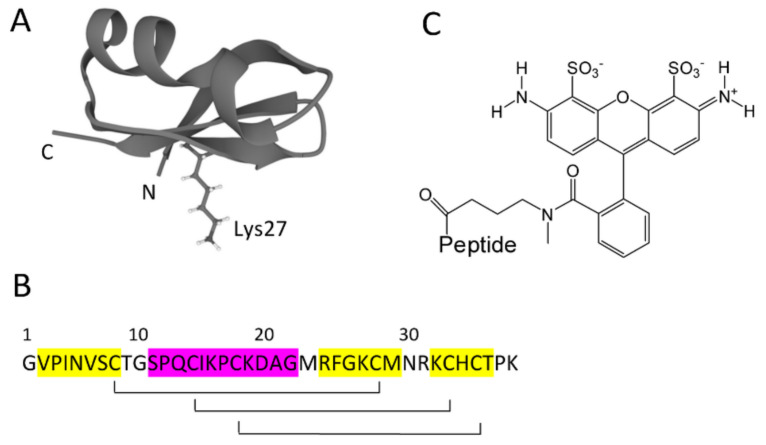
Structure of AgTx2 and Atto488. (**A**) Secondary structure of AgTx2 (PDB ID: 1AGT). The residue Lys27 that occludes a channel pore is shown in a stick-and-ball presentation. N and C denote the N- and C- termini of AgTx2. (**B**) Amino acid sequence of AgTx2 and distribution of secondary structure elements (magenta—residues of α-helix, yellow—residues of β-strands). Brackets connect disulfide-bonded cysteine residues. (**C**) Structure of Atto488 fluorophore.

Agitoxin 2 (AgTx2, Figure 1A,B), a 38-aa peptide toxin, was first isolated from *Leiurus quinquestriatus* var. hebraeus venom and characterized as a high-affinity inhibitor of the Shaker K^+^ channel and the mammalian voltage-gated potassium channels Kv1.x (x = 1, 3, 6) [1]. The tertiary structure of the toxin resolved by NMR spectroscopy [2] showed that secondary structure elements comprise more than 80% of the AgTx2 sequence (Figure 1A,B). The toxin contains a triple-stranded antiparallel β-sheet with two outer strands—S1 (Val2-Cys8) and S2 (Arg24-Met29)—and the central strand S3 (Lys32—Thr36). Strands S1 and S2 are connected by a helical region (Ser11—Gly22), which is located above the plane of the β-sheet. A characteristic cysteine-stabilized α-helix/β-sheet fold of the polypeptide chain is maintained by three disulfide bonds (Figure 1B).

**Figure 2:**
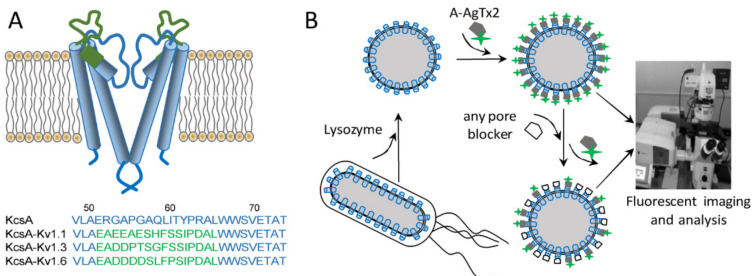
Hybrid KcsA–Kv1.x (x = 1, 3, 6) channels and their application in the analytical bioengineering system for the study of Kv1 channel blockers. (**A**) Structure of the hybrid KcsA–Kv1.x (x = 1, 3, 6) channels. Two of four channel subunits are shown for clarity. The green color in the structure and sequences indicates the blocker binding site of eukaryotic Kv1.x channels that was transferred to the KcsA channel. (**B**) A principle of the KcsA–Kv1.x (x = 1, 3, 6) channel application in the analytical bioengineering system. Hybrid channels expressed in the internal membrane of *E. coli* cells become accessible for interaction with the fluorescently labeled ligand A-AgTx2 after the lysozyme-mediated removal of the cell wall and the transformation of cells into spheroplasts. Binding of A-AgTx2 as well as the competitive binding of non-fluorescent pore blockers to KcsA–Kv1.x (x = 1, 3, 6) are detected and quantified using a laser scanning confocal microscope.

AgTx2 is widely used in the structural studies of K^+^ channels. The rigid tertiary structure of the toxin serves as a calliper to estimate the geometry of the toxin binding site, which is located on the outside of the Kv1 channel pore [2,3]. AgTx2 and its mutant forms have assisted in the studies of the location of the selectivity filter of the K^+^ channel and the molecular determinants that mediate the high-affinity interactions of K^+^ channels with pore blockers [3,4,5]. Radiolabeled and fluorescently labeled derivatives of AgTx2 have been found to be useful in combination with hybrid KcsA–Kv1.x channels (Figure 2A) for the study of pore blockers in animal venoms [6].

The design of bioengineered hybrid KcsA–Kv1 channels is supported by the findings that (i) the structure of the bacterial potassium channel KcsA is very similar to the structure of the pore domain of eukaryotic K^+^ channels [7], and (ii) the replacement of the residues in the P-loop of KcsA with the corresponding residues of eukaryotic Kv1.x channels creates high-affinity ligand binding sites of eukaryotic channels in KcsA [8,9]. KcsA–Kv1.x hybrids have facilitated studies on the interaction between the peptide blockers and the Kv1 channels’ binding sites, thus offering a new approach for the research related to K^+^ channel pharmacology [6].

Since fluorescently labelled AgTx2 derivatives retain nanomolar affinity for the Kv1.x channels (Ref. [6] and references therein), AgTx2 can be used as a template suitable for the design of novel fluorescent probes for the study of Kv1 channels. Currently, the choices for such probes are limited, although there is growing interest in in vitro and in vivo fluorescence imaging [10]. AgTx2 labeled with carboxytetramethylrhodamine (R-AgTx2) at Lys38 was shown to possess nanomolar affinity for the binding sites of the Kv1.x (x = 1, 3, 6) channels [11,12,13]. High-affinity and genetically encoded fluorescent ligands, in which a fluorescent protein (RFP or GFP) was fused N- or C-terminally to AgTx2, were constructed, and an efficient procedure of their production in the *E. coli* expression system was developed [14,15]. RFP-AgTx2 was shown to retain the pharmacological profile of AgTx2 for the Kv1 channels [14]. Unexpectedly, GFP-tagged AgTx2 ligands showed different affinities for the target channels depending on the position of the fluorescent tag. Whereas AgTx2 with a GFP tag at the C-terminus (AgTx2-GFP) interacted with each of the Kv1.x (x = 1, 3, 6) binding sites, the N-terminally labeled GFP-AgTx2 was found to interact with the Kv1.3 site only [15]. The enhanced selectivity of GFP-AgTx2 for the Kv1.3 site was supposed to be a result of intramolecular interactions between GFP and AgTx2 moieties, which masked AgTx2 residues important for the binding to the Kv1.1 and Kv1.6 channels.

To further clarify the effect of AgTx2 modification at the N-terminus, we aimed to evaluate the influence of a small organic fluorophore attached to the N-terminus of AgTx2 on the affinity and selectivity of ligand interaction with the Kv1 channels. For this, we studied AgTx2 conjugated with Atto488 dye through the N-terminal amino group (A-AgTx2, Figure 1C). We found that A-AgTx2 retained nanomolar affinity for the Kv1.3 binding site and lost its ability to interact with the Kv1.1 and Kv1.6 binding sites. A-AgTx2 was demonstrated to compete with peptide and low-molecular-weight blockers of the Kv1.3 channel for the binding to the Kv1.3 site, and it can be used as a component of the bioengineering analytical system based on the KcsA–Kv1.3 hybrid channel to study outer-pore blockers of the Kv1.3 channel.

## 2. Materials and Methods

### 2.1. Peptide Toxins and GFP-Tagged AgTx2

A-AgTx2 was synthesized by Smartox Biotechnology (Saint Egrève, France) with a purity of 99%, and it was used as received. Concentration of A-AgTx2 was measured by spectrophotometry using the molar extinction coefficient ε(500 nm) = 90,000 M^−1^ cm^−1^.

Recombinant peptides agitoxin 2 (AgTx2), kaliotoxin-1 (KTx1), charybdotoxin (ChTx), and scyllatoxin (ScyTx) were produced, as described earlier [16]. Concentrations of peptides were measured using molar extinction coefficients at 214 nm (49,200 M^−1^ cm^−1^ for AgTx2, 49,300 M^−1^ cm^−1^ for KTx1, 282,000 M^−1^ cm^−1^ for ChTx, and 41,600 M^−1^ cm^−1^ for ScyTx) that were calculated according to [17].

Fluorescent protein-toxin chimera AgTx2-GFP, which was tagged C-terminally with eGFP, was obtained according to [15]. Concentration of AgTx2-GFP was measured using ε(490 nm) = 55,000 M^−1^ cm^−1^.

### 2.2. Preparation of Spheroplasts

*E. coli* spheroplasts expressing KcsA or chimeric KcsA–Kv1.x (x = 1, 3, 6) channels were prepared according to the previously developed protocol [11]. Briefly, *E. coli* BL21 (DE3) cells (Novagen, Merck, Darmstadt, Germany), transformed either with the pET28a-KcsA, the pET28a-KcsA–Kv1.1, or the pET28a-KcsA–Kv1.3 plasmid, were cultivated in a minimal M9 medium at 37 °C up to OD_560_ = 0.8 optical units per cm (o.u./cm), induced with 50 μM isopropyl β-D-1-thiogalactopyranoside, and further grown at 37 °C for 20 h. The cells transformed with pET28a-KcsA–Kv1.6 were grown at 25 °C for 28 h after induction. To prepare the spheroplasts, cells were harvested by centrifugation (5000× *g*, 10 min, 4 °C), incubated in buffer A (10 mM Tris–HCl, pH 8.0, 0.5 M sucrose) containing lysozyme (20 μg/mL) for 7 min on ice, then diluted twofold with buffer B (10 mM Tris–HCl, pH 8.0, 0.3 mM ethylenediamine tetraacetic acid) and further incubated for 20 min. Finally, the spheroplasts (OD_560_ ≈ 0.5 o.u./cm) were stabilized by the addition of MgCl_2_ (10 mM) and stored at 4 °C. To obtain spheroplasts from non-transformed cells, *E. coli* BL21 (DE3) cells were subjected to similar growth, induction, and treatment procedures.

### 2.3. Microscopy

All the experiments were performed in a buffer containing 50 mM Tris-HCl (pH 8.0), 4 mM KCl, 50 mM NaCl, 10 mM MgCl_2_, 0.3 mM EDTA, 250 mM sucrose, and 0.1% bovine serum albumin. In the binding experiments, the spheroplasts (1000 cells/μL) were incubated with different concentrations of A-AgTx2 (0.16–20 or 100 nM) or AgTx2-GFP (4 or 50 nM) for 1 h at 37 °C. In the competitive binding experiments, the spheroplasts were incubated with A-AgTx2 (10 nM) and with one of the following compounds for 1 h at 37 °C: AgTx2 (0.4–50 nM), KTx1 (0.04–2.5 nM), ChTx (10 nM), ScyTx (5 µM), tetraethylammonium (TEA, 10 mM).

After incubation with ligands, the spheroplasts were placed in a 12-well flexiPERM silicon chamber (Perbio, Aalst, Belgium) attached to a thin (0.15 mm) cover glass and were centrifuged at 200× *g* for 5 min to immobilize the spheroplasts to a cover glass. The microscopy studies were carried out with a laser scanning confocal microscope LSM 710 (Zeiss, Oberkochen, Germany) using the α Plan-Apochromat 100×/1.46 oil immersion objective. Fluorescence of A-AgTx2 and AgTx2-GFP was excited at the 488 nm wavelength and recorded in the 495–590 nm range, at a 0.25 μm lateral and ca. 1.5 μm axial resolution. Image J software (National Institutes of Health, Bethesda, MD, USA) was used to treat the images and define the fluorescence intensity of the ligand bound to each measured spheroplast and the average fluorescence intensity *I_a_* of the bound fluorescent ligand (mean ± SEM, n = 130–230 cells per point).

The dissociation constants (*K_d_*) of complexes between A-AgTx2 and KcsA–Kv1.3 were defined by fitting the experimental dependencies of *I_a_* onto the concentration *L* of A-AgTx2 with the following equation:

*I_a_* ([*L*]) = *I_as_* [*L*]/(*K_d_* + [*L*]) (1)

where *I_as_* is the *I_a_* value when the A-AgTx2 binding is saturated.

The apparent dissociation constant (*K_ap_*) of non-labeled ligands (AgTx2, KTx1) was calculated using the data of competition binding experiments according to the procedure described elsewhere [11] with the following equation:

*K_ap_* = *IC*_50_/(1 + [*L*]/[*K_d_*]) (2)

where [*L*] is a concentration of A-AgTx2 (10 nM), *IC*_50_ is the concentration of a non-labeled ligand that displaces 50% of A-AgTx2 from the complex with KcsA–Kv1.3. *K_ap_* was introduced instead of *K_d_* to emphasize an indirect method (the competitive binding experiment) for its determination.

The *K_d_* and *K_ap_* values from three independent measurements were averaged and presented as mean ± SEM.

## 3. Results

### 3.1. Interaction of A-AgTx2 with Ligand-Binding Sites of Kv1.x (x = 1, 3, 6) Channels

A-AgTx2 was produced by solid-phase chemical synthesis that allowed one to get a fluorescent derivative of the peptide with an N-terminal position of a fluorophore. To characterize the interaction of A-AgTx2 with the pore blocker binding sites of the Kv1.x (x = 1, 3, 6) channels, hybrid KcsA–Kv1.x (x = 1, 3, 6) channels were used, which were presented at the surface of *E. coli* spheroplasts (Figure 2). Previously, it was demonstrated and independently confirmed in electrophysiological studies that these hybrid channels allow for the reliable recognition of Kv1-channel pore blockers and an estimation of their affinities to the binding sites of eukaryotic Kv1 channels [6].

**Figure 3:**
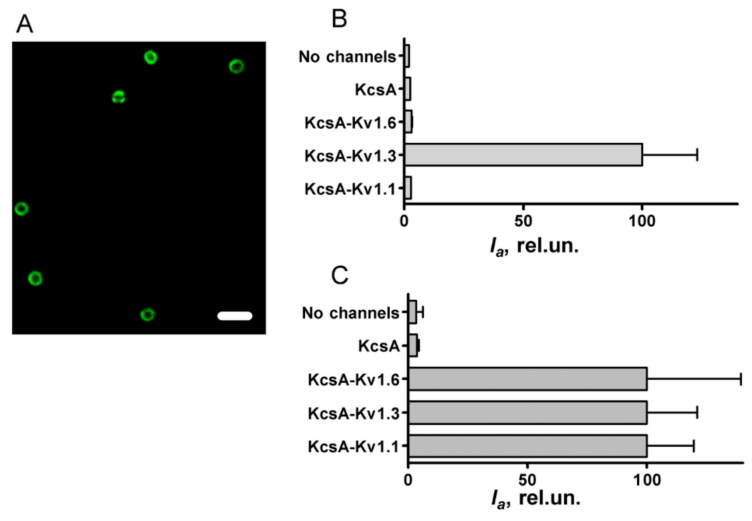
Study of the binding of A-AgTx2 and AgTx2-GFP to hybrid channels KcsA–Kv1.x (x = 1, 3, 6). (**A**) Typical confocal fluorescent image of A-AgTx2 (20 nM) bound to KcsA–Kv1.3 at the surface of spheroplasts. Bar is 2 µm. (**B**) Average fluorescence intensity *I_a_* of A-AgTx2 bound to spheroplasts expressing each of the KcsA–Kv1.x (x = 1, 3, 6) channels and to control spheroplasts prepared from KcsA-expressing or non-transformed cells. Concentration of A-AgTx2 was 20 nM in the case of KcsA–Kv1.3 and 100 nM in other cases. (**C**) Average fluorescence intensity *I_a_* of AgTx2-GFP bound to spheroplasts expressing each of the KcsA–Kv1.x (x = 1, 3, 6) channels and to control spheroplasts. Concentration of AgTx2-GFP was 4 nM in the cases of KcsA–Kv1.3 and KcsA–Kv1.6; in other cases, it was 50 nM. The *I_a_* values are the means of three independent experiments (mean ± SEM, *n* = 3).

It was found that A-AgTx2 effectively binds to spheroplasts expressing KcsA–Kv1.3 but does not stain spheroplasts presenting KcsA–Kv1.1 or KcsA–Kv1.6 (Figure 3A,B).

The intensity of the A-AgTx2 fluorescence detected from KcsA–Kv1.3-bearing spheroplasts was fifty times higher than that from control spheroplasts, i.e., spheroplasts expressing KcsA or not expressing heterologous channels. This difference was preserved even if control spheroplasts were incubated at an A-AgTx2 concentration fivefold higher than the KcsA–Kv1.3-bearing spheroplasts (Figure 3B). This means that A-AgTx2 binding occurred in the Kv1.3 binding site of KcsA–Kv1.3, while non-specific ligand binding to the KcsA channel scaffold or plasma membrane was very low.

Considerable levels of KcsA–Kv1.1 and KcsA–Kv1.6 presentation in the plasma membrane as well as the ability of these channels to bind suitable pore blockers were confirmed in experiments with AgTx2-L3-GFP (Figure 3C). In accordance with the previously reported properties [15], AgTx2-L3-GFP intensely stained spheroplasts presenting KcsA–Kv1.1, KcsA–Kv1.3, or KcsA–Kv1.6 but not control spheroplasts (Figure 3C). Therefore, the similarly low intensity of A-AgTx2 fluorescence recorded from control spheroplasts and spheroplasts bearing the KcsA–Kv1.1 or KcsA–Kv1.6 channels (Figure 3B) can be explained by the inability of A-AgTx2 to interact with the Kv1.1 and Kv1.6 binding sites.

We conclude that in contrast to non-modified AgTx2, A-AgTx2 is characterized by considerably increased selectivity toward Kv1.3 as compared to the Kv1.1 and Kv1.6 binding sites.

### 3.2. Properties of A-AgTx2 as a Fluorescent Probe in the Bioengineering Ligand-Binding System with KcsA-Based Hybrid Channels

**Figure 4:**
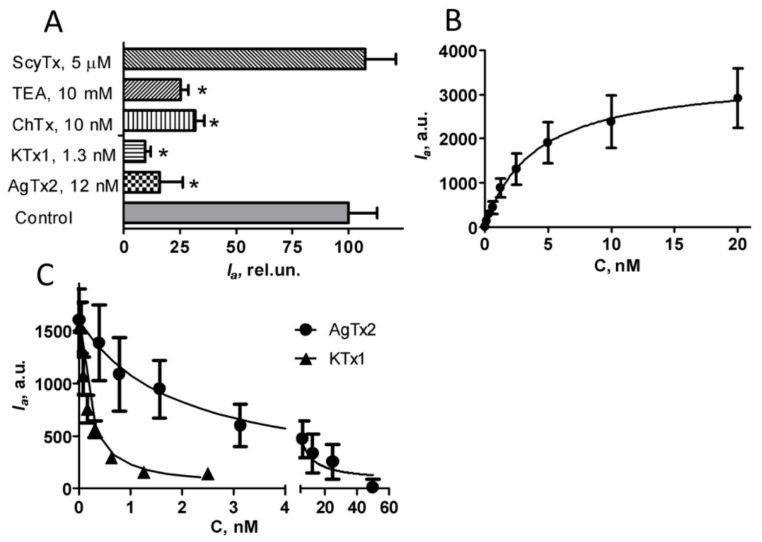
Interactions of A-AgTx2 and non-labeled peptide blockers with the Kv1.3 binding site. (**A**) Displacement of A-AgTx2 (10 nM) from the complexes with KcsA–Kv1.3 by AgTx2, KTx1, ChTx, TEA, and ScyTx. *I_a_* is an average fluorescence intensity of A-AgTx2 bound to spheroplasts expressing KcsA–Kv1.3 (mean *±* SEM is shown; sampling size was >390 cells per point. The results were averaged over 3 independent experiments; *—*p* < 0.01). (**B**) Typical concentration dependence of the A-AgTx2 binding to KcsA–Kv1.3 at the membrane of spheroplasts (mean *±* SEM is shown, sampling size is >130 cells per point). (**C**) Typical concentration dependences of the displacement of A-AgTx2 (10 nM) from the complexes with KcsA–Kv1.3 by AgTx2 and KTx1 (mean *±* SEM is shown, sampling size is >130 cells per point).

Interactions of A-AgTx2 with the Kv1.3 binding site were further characterized using known small organic molecule (TEA) and peptide blockers (ChTx, KTx1, AgTx2) of the Kv1.3 channel. The binding of A-AgTx2 to KcsA–Kv1.3 was shown to be responsive to the presence of Kv1.3 pore blockers (Figure 4A). High–affinity peptide blockers (ChTx, KTx1, AgTx2) displaced A-AgTx2 from the complexes with KcsA–Kv1.3 at nanomolar concentrations, while low–affinity TEA accomplished this at millimolar concentrations, as expected. A control peptide ScyTx, which blocks the KCa2.1, KCa2.2, and KCa2.3 channels [18] and does not interact with Kv1.3, did not disturb the complexes of A-AgTx2 with KcsA–Kv1.3 (Figure 4A). Therefore, a bioengineering system based on KcsA–Kv1.3-bearing spheroplasts and A-AgTx2 can be used to reveal the outer pore blockers of the Kv1.3 channel among peptides and small organic molecules in a convenient mix-and-read format.

The concentration dependence of A-AgTx2 binding to KcsA–Kv1.3-bearing spheroplasts was measured to estimate the dissociation constant (*K_d_*) of the formed complexes, as described in Section 2 (Figure 4B). *K_d_* value was found to be 3.9 ± 0.2 nM (mean ± SEM, *n* = 3), thus demonstrating that A-AgTx2 is a high-affinity ligand.

Since the bioengineering system based on KcsA–Kv1.3-bearing spheroplasts is suitable for the quantitative analysis of the affinity of unlabeled pore blockers by measuring the competitive binding of the fluorescent ligand and the compound under study [11], we tested A-AgTx2 in such an analysis (Figure 4C). AgTx2 and KTx1 demonstrated the concentration-dependent displacement of A-AgTx2 from the complexes with KcsA–Kv1.3 (Figure 4C). The concentrations of AgTx2 and KTx1 displacing 50% of A-AgTx2, which were determined from the measured dependences, made it possible to calculate the apparent dissociation constants of complexes (*K_ap_*) for the ligands, as described in Section 2. *K_ap_* values were equal to 0.54 ± 0.11 nM and 0.06 ± 0.02 nM for AgTx2 and KTx1, respectively. These values are consistent with those estimated previously using KcsA–Kv1.3-bearing spheroplasts and the GFP-tagged AgTx2 ligand (0.23 and 0.1 nM, respectively) [15], and they are in general accordance with the data of the electrophysiological studies reporting a *K_d_* of 2 nM for AgTx2 [19] and 0.1 nM for KTx1 [20]. Thus, A-AgTx2 can be used for both the recognition and the quantitative analysis of Kv1.3 pore blockers.

## 4. Discussion

The family of Kv1 channels is presented by homologous members Kv1.1–Kv1.8, among which the Kv1.1, Kv1.2, and Kv1.6 channels are known to control neuronal excitability in the central nervous system, while Kv1.3 is a prominent regulator of T lymphocyte activation [21] and a recognized therapeutic target [22].

Peptide pore blockers, particularly those found in scorpion venoms, are considered as perspective agents that can exert a therapeutic effect by inhibiting Kv1 activity. However, natural peptide blockers are active against several closely related Kv1 channels, and only a few of them are characterized by a high selectivity for the target channel; for example, the Kv1.3 channel blocker Vm24 from *Vaejovis mexicanus smithi* [23] and the Kv1.2 channel blocker MeKTx11-1 from *Mesobuthus eupeus* [24]. Usually, the mutagenesis of natural peptides is used to increase the selectivity of the peptide blockers of Kv1 channels [25,26,27]. In the present study, the selectivity of AgTx2 for the Kv1.3 binding site was achieved by attaching a small fluorophore Atto488 to the N-terminus of the peptide.

Our previous experience shows that a small organic dye, for example, carboxytetramehylrhodamine, can be conjugated to AgTx2 without changes in the binding profile of the conjugate to the Kv1 channels [11,12,13]. Differences between two labeled derivatives include the position of a label (carboxytetramehylrhodamine was located at the C-terminus of AgTx2 [11]), the number of charges, and the total charge at the labels. Atto488 bears two negatively and one positively charged groups (Figure 1C), while carboxytetramehylrhodamine bears one negative charge and one positive charge. It can be supposed that the N- vs. C-terminus position of a label is the most important factor, since AgTx2 also demonstrated a loss of affinity to the Kv1.1 and Kv1.6 binding sites when the GFP tag was attached to the N-terminus of AgTx2 [15]. However, this conclusion is not unambiguous, since AgTx2 with RFP at the N-terminus preserved the binding to the Kv1.1, Kv1.3, and Kv1.6 channels [14]. Since GFP and RFP differ considerably in the surface charge (−7 and −1, respectively), we can conclude that two factors contributed to the observed selectivity: the N-terminal position of a substituent and its negative charge. Of course, the spatial localization of the negative charge(s) can also be important in efficiently disturbing the ligand binding to Kv1.1 and Kv1.6.

The effect observed for the AgTx2 derivatives is quite similar to that reported for the sea anemone toxin ShK, which in its natural form has a high affinity to the Kv1.3, Kv1.1, Kv1.4, and Kv1.6 channels [28]. Synthetic derivatives of ShK modified at the N-terminus with phosphotyrosine or its analogs acquired pronounced selectivity for the Kv1.3 channel over closely related channels [29,30].

The most exposed part of the Kv1 binding site contains an array of anionic amino acid residues in each of the subunits (EEAE in Kv1.1, DDPT in Kv1.3, DDDD in Kv1.6) [13,15], which may partially restrict the access of ligands with negatively charged groups to the binding site, especially in the cases of Kv1.6 and Kv1.1. Moreover, the total negative charge of the binding site increases in the row Kv1.3 (−16) < Kv1.1 (−20) < Kv1.6 (−24) [13]. Negative charge(s) at the N-terminus of the AgTx2 derivatives can potentially interfere with some negatively charged groups in the binding sites of Kv1.1 and Kv1.6 and thus hamper the formation of complexes.

Definitely, extended studies are needed to clarify the influence of the structure and charge of the fluorescent label attached to the N-terminus of AgTx2 on the binding profile of the conjugate with Kv1 channels. The structural diversity of available fluorescent labels and technology in a solid-phase peptide synthesis will provide us with a set of AgTx2 derivatives that can be used to resolve this issue in further research.

As shown in the present study, the properties of A-AgTx2 allows one to use it as a component of the bioengineering analytical system based on the hybrid KcsA–Kv1.3 channels for the search and study of Kv1.3 blockers. A-AgTx2 can also be considered as a high-affinity fluorescent probe for the imaging of the Kv1.3 channel in cells and tissues, but it requires a special study.

The results of our study demonstrate that the Atto488 label introduced two new functionalities to AgTx2: fluorescence and selectivity of high-affinity binding to the Kv1.3 channel. Here, fluorescent labeling served as an alternative to conventional site-directed mutagenesis aimed at changing a pharmacological profile of the channel blocker. One can assume that by introducing different fluorescent labels into some peptide blockers of Kv1 channels, both the color diversity of Kv1-channel fluorescent probes and a modified spectrum of pore blocker activities can be achieved.

## Data Availability

The data presented in this study are available on request from the corresponding author. The data are not publicly available due to local regulations.
